# The case for studying the intergenerational transmission of social (dis)advantage: A reply to Gary Marks

**DOI:** 10.1111/1468-4446.12813

**Published:** 2021-01-15

**Authors:** Bastian A. Betthäuser, Erzsébet Bukodi, Mollie Bourne

**Affiliations:** ^1^ Nuffield College University of Oxford Oxford UK; ^2^ Department of Social Policy and Intervention University of Oxford Oxford UK; ^3^ Engineering UK London UK

Our article “Understanding the mobility chances of children from working‐class backgrounds in Britain: How important are cognitive ability and locus of control?” examines the role of cognitive ability and peoples’ sense of control over their lives in mediating the effects of individuals’ social background on their educational attainment and on their labor market position (Betthäuser et al., 2020a). The article takes as its starting point the persistent view in both academic and policy circles that most of the differences in the educational attainment and labor market success between individuals from different socio‐economic backgrounds are due to differences in cognitive ability between them (see e.g., Marks, 2014; Murray, 2012). Using data from the 1970 British Birth Cohort Study,^1^ we find that cognitive ability mediates a non‐negligible yet limited amount of the effect of individuals’ social background on their educational attainment (about 35%) and their labor market position (about 20%). This means that about 65% of the effect of individuals’ social background on their educational attainment, and about 80% of the effect on their labor market position, is channeled by factors *other than* cognitive ability. Contradicting the claims by Murray (2012), Marks (2014), and others, this finding highlights that the intergenerational reproduction of social (dis)advantage that prevails in even the most developed societies is deeply unmeritocratic and unfair. Consequently, we see an urgent need for researchers to identify and for policy makers to address the channels through which individuals’ parental class background shapes their life chances, above and beyond its effects on individuals’ cognitive ability.

In his commentary on our article, Gary Marks (2020, p. 3) concludes that the findings of our article “are technically correct but unimportant.” He argues that examining the role of cognitive ability in mediating the association between individuals’ social background and their educational and labor market outcomes is not a relevant exercise, since there are “only moderate associations of class origins with educational and occupational outcomes” (p. 2). Instead, he suggests that research should focus on the importance of individuals’ genetic predisposition and cognitive ability in affecting individuals’ educational attainment and labor market outcomes (p. 2). In short, Marks takes issue, not with the substance and the findings of our article, but with the research question we pose and with our motivation for addressing it.^2^ ^,^ ^3^ Our reply, therefore, focuses on why, in our view, it is imperative for social scientists across different disciplines to critically examine the association between individuals social background and their educational and labor market outcomes, and to understand the role of different factors—including cognitive ability—in accounting for this association.

We strongly disagree with the claim by Marks that there are “only moderate associations of class origins with educational and occupational outcomes” (p. 2). Research on social stratification and mobility in sociology, economics, and psychology has demonstrated that individuals’ social background continues to yield a strong influence on both their educational and labor market chances (Bukodi and Goldthorpe, 2018; Chetty et al., 2014; Laurison & Friedman, 2016; Major & Machin, 2018; Von Stumm et al., 2009). To use the analogy of John Rawls (1971), the ticket that people draw in the “lottery of birth” continues to matter for their life chances. This is true even in the most democratically and economically advanced societies, such as Britain. To illustrate this, Figure 1 shows the extent to which individuals’ social background affects their educational attainment. More specifically, it depicts the chances of individuals with the *same level of cognitive ability* but from *different social backgrounds* to attain an upper secondary or a higher level of qualification in Britain. We show this separately for women (right panel) and for men (left panel) and for different birth cohorts, spanning the last five decades. Individuals are split into three groups, based on their parents’ social class, social status, and educational attainment. The most advantaged group (10% in the earliest cohort and 27% in the most recent cohort) are predominantly the children of parents in the managerial and professional salariat or at least in white‐collar occupations who have tertiary‐ or at least upper secondary‐level qualifications. The least advantaged group (50% in the earliest cohort and 30% in the most recent cohort) are predominantly the children of parents in wage‐earning, mainly blue‐collar occupations with no qualifications or at best only ones at a lower secondary level. What this figure clearly shows is that individuals’ social background significantly shapes their educational attainment and that this is so even when we compare people who have the *same level of cognitive ability*. By way of example, a woman with an intermediate level of cognitive ability born in 1990, who comes from the most advantaged social background is, on average, about forty percentage points more likely to attain a qualification at the upper‐secondary level or above, compared to a woman with the same level of cognitive ability who comes from the least advantaged social background. For men, this difference is even more pronounced. There is no doubt that this constitutes a very substantial effect on individuals’ social background on their educational chances. And notably, this gap in the educational attainment of people coming from different social backgrounds has persisted over time. To claim that there are only moderate associations between peoples’ social origins and their educational and occupational outcomes, as Marks does, is, therefore, a misrepresentation of the evidence. To suggest that this association does not warrant further research is unfounded and neglects the responsibility of researchers to focus on what in our view is one of the key challenges that societies continue to face: to equalize the highly unequal playing field faced by individuals from different social backgrounds.

**FIGURE 1:**
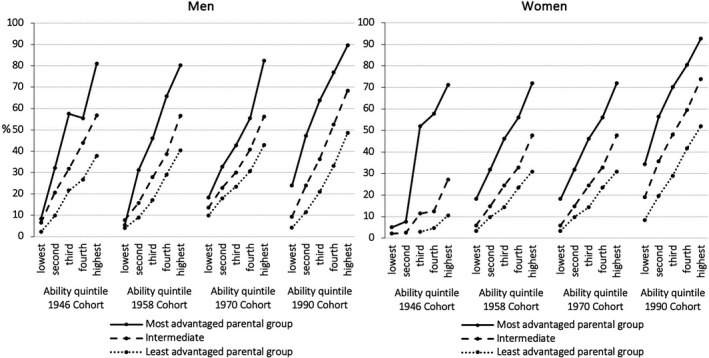
Estimated probabilities (%) of attaining upper secondary or higher level of qualification by parental group, cognitive ability quintile and cohort

The substantial gap in life chances between people from different social backgrounds is problematic both from a normative perspective and from an efficiency point of view. Seen from a normative perspective, it is socially unjust if individuals’ life chances depend on ascriptive characteristics, which are out of their control, such as the social circumstances into which they are born (Rawls, 1971). From an efficiency perspective, it is undesirable that individuals’ educational attainment and the type of job they have depends on their family background, rather than their ability and skills (Betthäuser, 2017; Gray and Moshinsky, 1935). In contrast to what Marks claims, these are important grounds for why it should be a top priority for researchers across different disciplines to study how and why peoples’ social background continues to exert such a strong effect on their life chances. Examining, as we do in our article, the extent to which the gap in the educational attainment and labor market success between individuals from different socio‐economic backgrounds are due to differences in cognitive ability between them is one important step toward this larger goal.

In his commentary, Marks claims that we “misrepresent the research [we] take issue with” (p. 2). Here we would like to ask the reader to examine the arguments made by Murray, Marks, and others, which we set out to test empirically in our article. As we note in the article, Murray argues that “the reason that upper‐middle‐class children dominate the population of elite schools is that the parents of the upper‐middle class now produce a disproportionate number of the smartest children” (2012, p. 60). Murray suggests that this further explains why individuals in advantaged labor market positions largely come from higher social class backgrounds (2012, pp. 46–68). He also posits that the transmission of intelligence is largely genetic and is reinforced by increasing homogamy, that is, the growing tendency of people to form partnerships with individuals of similar social standing (2012, pp. 46–68). These views are echoed by a number of sociologists and social psychologists (see, e.g., Gottfredson, 2003; Marks, 2014; Plomin, 2018; Saunders, 1997, 2012). With regards to the effect of individuals’ social background on their educational attainment, for instance, Marks (2014, p. 88) writes that “the inclusion of [cognitive] ability in the analysis reduces the impact of socioeconomic background considerably and in some cases to statistical insignificance.” With respect to the effects of individuals’ social background on occupational and economic outcomes, he further contends that “the direct impact of socioeconomic background is even smaller, and smaller again after taking into account educational attainment and, to a lesser extent, cognitive ability” (Marks, 2014, p. 234). These arguments clearly advance the claim that the differences in the educational attainment and labor market success between individuals from different socio‐economic backgrounds are due to differences in cognitive ability between them.

We believe that it is important to empirically test the arguments made by Marks and others, particularly because of their political potency. A large mediating role of cognitive ability can be (mis‐) interpreted to imply that the pronounced inequality in educational and labour market attainment between individuals from different social backgrounds is somehow efficient or legitimate and does not require political intervention. For example, as we note in our article, Dominic Cummings, who was the special advisor to the British Secretary of State for Education, Michael Gove, and Chief Special Advisor to the Prime Minister, Boris Johnson, contends that “differences in educational achievement are not mainly because of ‘richer parents buying greater opportunity’” and suggests that they are instead due to richer parents having more capable children than poorer parents (Cummings, 2013, p. 74). Cummings further argues that any policy that aims at equalizing educational opportunities and increasing the quality of education would *increase* the effect of children's social background on their educational achievement, thereby questioning the importance of lowering the effect of children's social background on their educational attainment as a policy priority (ibid.). In our article and our related work, we show that these arguments are unfounded and that equalising educational opportunities can substantially *reduce* the effect of children's social background on their educational achievement and labour market chances (Betthäuser, 2017; Betthäuser et al., 2020a).

In sum, the evidence clearly shows that individuals’ social background continues to exert a strong influence on their life chances. Understanding how and why this occurs is of utmost importance from a scientific, from a normative, from efficiency, and from a policy point of view. As a step toward this larger goal, our article focuses on examining the extent to which differences in cognitive ability account for the gap in educational and labor market attainment between individuals from different social backgrounds. We find that cognitive ability plays a relatively modest role—a far more limited one than the Murray (2012), Marks (2014), and others suggest—in accounting for the substantial gap in life chances between individuals from different social backgrounds. Clearly, this raises the question through which other ways peoples’ social background continues to shape their life chances. We urge researchers from all disciplines to improve our understanding of this question through careful empirical analysis. Moreover, we believe that it is the responsibility of policy makers from across the political spectrum to use this knowledge to level the highly inequitable playing field that people from different social backgrounds continue to face.

## ENDNOTES

^1^ For further information on the 1970 British Birth Cohort Study and an in‐depth description of the variables and measures we use in our analyses, please see Betthäuser et al. (2020b).

^2^ On a more technical note, Marks observes that in our results “the figure of 35% for [the part of the social background effect mediated by cognitive ability] contrasts with the 50% estimate in a similar paper by Bukodi et al. (2019).” First, we would like to highlight that our earlier paper, which Marks refers to, finds that the percentage of the social background effect on educational attainment that is mediated by cognitive ability varies between 30% and 50%, depending on the specific birth cohort and the dimension of individuals’ social background considered. For the 1970 birth cohort, we find that about 30% of the parental class effect on individuals’ educational attainment is mediated by cognitive ability (Bourne et al., 2018, p. 16). The difference in the mediating role of cognitive ability that we find in this earlier paper, as compared to our present paper, is simply due to the fact that in our present paper—as stated in the title of the paper—we focus on the effect of coming from a working‐class background, rather than a more advantaged background. By contrast, our earlier paper examines the role of cognitive ability in mediating the effect of a more general measure of parental social class on educational attainment (Bourne et al., 2018, p. 14).

^3^ Marks also claims that we “underplay the importance of education for occupational destinations […]. Net of educational attainment, occupational origins have little impact on occupational destinations […].” In contrast, we clearly state that educational attainment, as operationalized in our analysis, accounts for 30%–40% of the association between parents and their children's social class position and that this depends on the social class cut‐off we examine (p. 359). This is in line with past research on Britain (Breen & Karlson, 2014), and with evidence from other societies, such as Germany (Betthäuser, 2020). There is now a growing literature that seeks to examine the ‘direct’ effect of individuals’ social class background on their labor market position that is not mediated by their educational attainment (see e.g., Bernardi & Ballarino, 2016).
